# Hospital and Institutional News

**Published:** 1915-01-23

**Authors:** 


### HOSPITAL AND INSTITUTIONAL NEWS.

### THE CHADWICK PRIZE FOR NAVY AND ARMY DOCTORS.

Dr. F. M. Sandwith, Chairman of the County of London Branch of the British Red Cross Society, as we report in full on page 375, delivered the first of three lectures on "War and Disease" at the Royal Society of Arts last week, on the subject of "Continental Wars of the Nineteenth Century." The occasion of the lectures, which are being delivered under the provisions of the Chadwick Trust, takes on an added interest from the announcement of Sir William Collins, Chairman of the Chadwick Trustees, that, mindful of the war, they had availed themselves of powers included in the Trust to offer a prize of fifty guineas and the Chadwick Gold Medal to the medical officers of the Navy and the Army who most distinguished themselves in promoting the health of our sailors and soldiers on active service. The war had provided much direct evidence of the reforming work done by Edwin Chadwick and Florence Nightingale. In dealing with the Continental wars of the past century, Dr. Sandwith devoted attention to the very serious results that had accrued in the past from the neglect of military sanitation and hygiene, and stated that the present endurance of severe physical conditions on the part of the British soldier was largely due to a condition of health for which the Royal Army Medical Corps, with its fine *personnel* and organisation, was responsible. A more fruitful subject than a study of mistakes made in the past and the consequences due to them could hardly be imagined. Even official departments, which are often slow to learn, are not always quick to remember, and with the great problems now pressing on us at home, particularly in relation to the camps of the new armies and the difficulties of providing proper hygienic conditions for the thousands of men for whom accommodation has had to be improvised, of late, too, under extremely trying conditions, the enforcement of past experience is most salutary in the lecture hall and the Press, as well as in the actual camps themselves.

### THREE MONTHS' CONTRACTS FOR POOR-LAW INFIRMARIES.

Many Boards of Guardians whose yearly contracts terminate in March will require to consider what arrangements they should make for the coming year's supplies. The question of contracts was dealt with in The Hospital of August 22, 1914, and, as we then advised, most boards have adopted the sensible course of meeting their contractors' claims by considering any extra charges to be made due to the war. The policy has been adopted of having goods invoiced at contract prices and at the end of a term submitting any extra prices to the responsible officials for their opinion before making payment. It may here be mentioned that in the majority of cases contractors have dealt with the matter in a spirit of fairness and have not unduly taken advantage of this method. In some cases, as at Edmonton, the question of extra payment for provisions, meat, etc., has been referred to the London Chamber of Commerce for their opinion as to the justness of the amounts claimed. As regards making new contracts, in view of the high prices obtaining for certain commodities, especially medical supplies, it is probably not desirable to place contracts for a long period, for if supplies become better owing to home production, these high prices will have to be paid throughout the year. Short contracts will also suit contractors, for, owing to the uncertainty of the period of duration of the war, they will be chary of long engagements without charging sufficiently high prices to cover them against probable loss. It will, therefore, most likely be necessary to have short contracts—say, three-monthly—during the war period for drugs, chemicals, and other articles which are liable to fluctuations.

### RIGHTS OF CIVILIANS AT HALIFAX ROYAL INFIRMARY.

The Halifax Health Insurance Committee, presided over by Mr. A. Culpan, considered last week a report from the house committee of the Halifax Royal Infirmary in reply to a resolution passed by the Insurance Committee on December 8 last. The resolution recommended the infirmary committee, in their loyalty and anxiety to care for wounded soldiers, not to diminish the hospital facilities for the insured person. With regard to certain cases of complaint alleged by Dr. Muir, he had furnished details of one to the infirmary committee, and stated, said their report, the others to be based on second-hand information. The report stated that their examination of the one case had led them to find the complaint based on it "without a shred of justification." In regard to Dr. Muir's specific complaint, the report of the infirmary committee described it as an incurable case, the retention of which would have defeated the purposes for which the institution was founded. "The suggestion," states the report, "that the request for the removal of the patient was because it was necessary to have beds for the wounded soldiers is absolutely unfounded . . . as on the day *before* the patient's discharge there were more vacant beds than there had been for a considerable time." After further discussion, it was decided to accept the infirmary's assurance that full consideration is given to all classes of cases.

### SOLDIERS IN HOSPITAL: AN OFFICIAL VIEW.

"If," concludes this section of the report of the Halifax Royal Infirmary on the question of soldiers in voluntary hospitals, "the question of receiving wounded soldiers had been decided on the financial considerations suggested, the soldiers would certainly not have been received. The Government allowance is about one-half of the cost per occupied bed, and the cases taken have been costly. But nothing could have been further from the truth than to suggest that the monetary consideration was in any degree the motive. The military authorities have been clearly and repeatedly informed that though the infirmary authorities are prepared to help in times of emergency, they do not desire to take the cases so long as the need does not exist. The demand for hospital accommodation for soldiers varies very greatly from time to time, but in the month of November the casualties were so numerous that not only were all the military hospitals crowded, but many general hospitals in the United Kingdom were also. If the boards of these latter had shown a mean and parochial spirit and had failed to realise their wider responsibility their hospitals might undoubtedly have been commandeered.'' Still another important aspect of this problem is referred to in the next paragraph.

### THE ALTERNATIVE TO HOSPITALS FOR THE WOUNDED.

We add to the above quotations the following paragraph of this most instructive report from the Halifax Royal Infirmary, with reference to the suitability of improvised hospital accommodation for the wounded. ''The suggestion that private houses with extemporised staffs are good enough for soldiers from the battlefields is one which the committee believe will meet with the indignation which it deserves. The military authorities decided at the outset that the soldiers must have at least as efficient treatment as civilians, but they afterwards, under pressure, tried the experiment of sending some cases to improvised quarters stated to be well equipped in every way. This experiment it was decided not to repeat. A very large proportion of the soldiers in the infirmary have required *x*-ray treatment, and many operations have been necessary.'' We have given these quotations at length because they form an historic document typifying in the case of one great voluntary hospital the position in which these institutions were placed on the outbreak of war, and the reasons which led them, or rather the circumstances which compelled them, to take the exceedingly onerous and difficult part they have in treating the wounded.

### A HOSPITAL SHIP FOR SERVIA.

The latest hospital ship is the *Erin*, belonging to Sir Thomas Lipton, who accompanied it on its departure from Southampton last Saturday *en route* for Salonica, carrying hospital equipment and stores for the Servians and Montenegrins. The ultimate destiny of the hospital will probably be Uskub or Nish, and the expedition, which is sent out jointly by the British Red Cross Society and the St. John Ambulance Association, will be under the control when in Servia of Captain E. N. Bennett, a Red Cross Commissioner, who has spent the last five months on active service in France and Belgium. The matron is Miss Callwell, lately in charge of the Sophia Berthelot Hospital at Calais. The expenses of the voyage have been defrayed by the owner of the *Erin*, who has also contributed to the supplies which it is taking out.

### A PATRIOTIC INSTITUTION.

At the recent quarterly court of the governors of the Royal Masonic Institution for Boys at Bushey, Herts, a notice of motion by Mr. Charles Edward Keyser was considered to admit under Law 68a six duly qualified boys ''as and from January 1, 1915, to the benefits of the institution, their fathers having been killed or died whilst on active service, and that they be and are hereby elected without ballot accordingly,'' subject to the regulations pertaining to boys after election. This patriotic action deserves every congratulation and encouragement, for such institutions only deserve the proud title of being philanthropic when they are ready to act in the spirit of their foundations, and, as in this case, show their practical appreciation of, and bear their part in, the service of the country, for which the fathers of these boys have given their lives.

### LONDON'S MENTAL HOSPITAL WORK.

Some interesting particulars are given by the London Mental Hospitals Committee in their annual report as to the growth of the work since the first committee was appointed in 1889. In that year there were four mental hospitals available for 7,270 patients, and one in course of construction for 2,000 patients. To-day there are ten mental hospitals available for some 20,300 cases, and in course of construction there are an eleventh hospital for more than 2,000 and the special institution which owes its inception to the generosity of Dr. Maudsley. At the end of 1889 the County of London was primarily responsible for just over 10,000 cases of insanity; at the end of 1913 the ascertained responsibility had risen to just under 21,200, an increase of approximately 110 per cent. in twenty-four years. In 1889 the mental hospital service numbered a staff of 1,070; to-day the staff is 4,028, and expenditure on the maintenance of patients has increased in round figures from £188,000 to £617,000.

### TRIBUTE TO A RED CROSS SECRETARY.

The Coventry Division of the British Red Cross Society at a meeting held last week gave a presentation to Mr. William Hutton in recognition of his services as secretary to the division. The gift, which took the form of a Sheraton chiming-clock, was presented by Colonel W. F. Wyley, who remarked that till lately they had tried in vain to get a detachment in the city, and now, thanks largely to Mr. Hutton's tact and ability, it only remained to know the result of the second examination and to get a few more men, and they could have a detachment in Coventry. In reply, Mr. Hutton said that when asked to become secretary of the division he accepted the honour knowing he would not be amongst those accepted to fight. When the war was over, he added, they must do what they could to remove the feeling of hatred between the opposed countries, and to bring about a settlement that would make this the last war. A significant tribute to the value of skilled secretarial work was paid by Dr. Brazil, who said that the work of the medical profession in taking classes, for which Colonel Wyley had thanked them so kindly, was small besides Mr. Hutton's, without whose lead the affair would have been a fiasco. Dr. Brazil also made a point about the presentation itself by reminding his audience how appropriate a Sheraton design was, since it was in vogue at the time of the battle of Waterloo. It is pleasant to see this mutual appreciation of medical and administrative work, on which all branches of hospital life depend, for it is not always so gracefully insisted on.

### THE WEST DERBY GUARDIANS AND MEDICAL SALARIES.

On the recommendation of the Aider Hey Hospital committee that Dr. Macdiarmid, senior medical officer to Walton Workhouse, be appointed visiting medical officer at a salary of £500 per annum, a lively discussion took place at a meeting of the West Derby Guardians last week. The Rev. J. W. Baker moved that the matter be referred back, as there was very strong feeling against the amount of salary proposed, on the ground that it would mean less work and a higher salary. At present Dr. Macdiarmid was receiving £400 per annum for the whole of his services at the workhouse. His proposed new position would involve less work, less time, and more pay. Dr. McCormack said that a severe medical famine would exist for years to come, and that next year, if the board wanted medical officers, they would have to pay more than at present. After Mr. D. J. Williams had remarked that the difficulty of public institutions was becoming daily greater, and that one in Liverpool he knew of was without a house surgeon, Dr. Grimes pointed out that this was promotion for the doctor, and that in Manchester £700 a year was being offered for a similar post. The voting was then taken, and resulted in eighteen being for and eighteen against Mr. Baker's amendment. The Chairman then gave his casting vote, also against it, and the original proposal was agreed to.

### THE FIRE AT DURSLEY INFECTIOUS HOSPITAL.

Early on Thursday last week what, but for the fine efforts of the small staff, would have proved a serious fire broke out at the Dursley Infectious Diseases Hospital, known as "The Moors," in the parish of Coaley. A room in the institution had been in course of disinfection, and throughout the day a big fire had been burning in the grate, before which a mattress was placed to be aired. On Wednesday night the housekeeper, Mrs. Hobbs, and the nurse, after assuring themselves that there was no danger, as the fire had apparently gone out, went to bed. Early, however, on Thursday morning the cry from one of the patients that he was choking awoke Mrs. Hobbs, who found her own room full of smoke. She hastily awakened the other occupants, including her husband and the nurse, who promptly removed the patients to comparative safety in another part of the institution. They then attacked the fire with buckets of water, each of which had to be obtained from a pump, and their efforts were fortunately aided by a man who happened to be about early and, seeing the smoke, came to their help. By this time a message was sent to the fire-station, but on the arrival of the brigade, soon afterwards, the fire had been got under control, though the floor, skirting, ceiling, and windows of the room were burnt out. Captain Talboys, of the brigade, declared that the stone stairway alone saved the building from destruction, and highly praised Mrs. Hobbs, her husband, and the nurse for all that they had done. As "The Moors" is an out-of-the-way place and in Mr. Hobbs's absence there is not a man on the premises, and but rarely more than one nurse, it will be seen how much depended upon the promptitude and energy of the small staff. It is not quite certain, on the evidence at present available, whether the mattress was the cause of the fire or not, but the moral is that in all institutions, particularly small ones, where, as here, the water supply is small in the extreme, precautions against fire must have regard not only to the diminution of all foreseeable risks, but also to the difficulties of getting assistance should an accident occur. If it is impossible to increase the water supply of a remote institution, a telephone to the nearest fire-station should always be installed.

### A TUBERCULOSIS DISPENSARY FOR MEN IN THE CITY.

The City of London Corporation, in connection with their dispensary for the treatment of tuberculosis at St. Bartholomew's Hospital for insured and uninsured patients, have notified the various Metropolitan Borough Councils that, in addition to actual residents in the City, the dispensary is available for any *bona fide* case who, though resident in other districts, may find it inconvenient or impossible to attend the dispensary established in their own district. No charge will be made for the services rendered, nor will the local authorities in which patients reside be asked to contribute towards the City's expenditure upon the dispensary. This offer of the City authorities has been accepted in the same generous spirit as that in which it is offered, and there is little doubt that the dispensary at St. Bartholomew's Hospital will become one of the most useful in the Metropolis.

### THE CASES AT CLAYBURY.

Dr. Robert Armstrong-Jones, the medical superintendent of Claybury Mental Hospital, in his annual report on the work of the institution, states that the average age of patients admitted was forty-two years in men and forty-four in women, and more married than single were admitted. In regard to the occupation of those admitted he states that insanity is found to be more frequent among those whose work is of a casual or of an uncertain nature, as also in those whose occupation tends to the more frequent use of alcohol. Those described as casual or general labourers, carmen, potmen, hawkers, porters, and commercial travellers are the most noted occupations, while clerks, both male and female, and persons of no occupation are also frequent. A female telephone operator referred her aural hallucinations to the constant strain involved in receiving and transmitting messages. Of the 494 patients admitted during the year a family history of insanity was ascertained to be an ætiological factor in 104 cases, or more than one-fifth of the whole; but the history was not always given, and when the history was full, an insane heredity, direct or collateral, was found in considerably more than one-half the cases. Among the factors of causation alcohol figured in ninety-three cases, a proportion which approximates to the average given for all the mental hospitals of England and Wales.

### THE PROFESSION AND MEDICAL TRADE UNIONISM.

The medical profession has lately been endeavouring to make up its mind whether or not to adopt frankly trade union principles, but so far with only very moderate unanimity. The only general agreement seems to be in some dissatisfaction with the British Medical Association, which has led certain members and non-members to favour a more militant policy. Thus, more than one new organisation on trade union lines has sprung into existence, the latest attempt in this direction being a new body formed to protect the interests of panel practitioners under the name of "The Panel Medico-Political Union," whose rules were submitted to a meeting of panel medical men at Caxton Hall last week. Dr. W. Coode Adams presided, and stated that they wished to run, if necessary, their own candidate for any seat in Parliament or for any Borough Council; and prided themselves on being essentially a trade union. Dr. Fothergill, however, moved that the rules be referred back for further consideration, as they ought to give another chance to the British Medical Association of doing what was desirable. This motion being carried, no further progress has so far been made, the old hesitation to supplement the British Medical Association with another purely trade union body once more asserting itself. Indeed, the moral seems to be that until the profession has succeeded in reconciling the different counsels which at present prevail, and the panel and non-panel interests, there is small chance at the moment of any new trade union body becoming sufficiently representative to have the necessary weight.

### THE "LUXURY" OF INSTITUTIONAL OFFICERS.

The Edmonton Guardians, at their last meeting in December, instructed the board's architect to prepare plans for a residence, in the grounds of the infirmary, for the medical superintendent, and, in addition, amended plans were to be prepared for a house on the suggested site near the Silver Street entrance. The cost was originally expected to be £2,000, but at the Guardians' last meeting it was decided to submit plans to the Local Government Board for a medical superintendent's residence at a cost of £1,200. Some opposition has been offered to this scheme, and at a meeting of the Ratepayers' Association held recently it was proposed to approach the Local Government Board with a view to preventing their official sanction. Many extravagant statements were made at this meeting, and also in the local Press, with the intention of creating the impression that the life of the institutional officer is one of luxury. Those who are engaged in institutional work know well that the medical superintendent of a large infirmary has to spend most of his life within its four walls, ready for any emergency, and especially at the present time, when there is a difficulty in obtaining suitable candidates for medical appointments, a comfortable and up-to-date residence must be provided for the chief medical officer and his family.

### A MINISTER AND THRIFT ON 8s. A WEEK.

There are occasions when the questions of Poor-Law relief become merged in those of public health, to which they are always allied, as was shown at last week's meeting of the Darlington Board of Guardians. In the course of the meeting it was stated that the Hartlepool Guardians had asked the Darlington Board to relieve on their behalf a woman and three children resident in the board's area. It was proposed to allow the woman 8s. per week. The clerk stated that the children were quite young, one being in arms, and that the woman had no other source of income. On it being moved that the Darlington Board decline to act as agents unless the Hartlepool Guardians raised the amount to 18s. a week, the Rev. John Clegg said that he thought the board had no right to take up such an attitude. They had not yet ascertained, he added, that the woman and her three children could not live on 8s. a week. The clerk's reply to this was, "Some facts are self-evident," and we are glad to see that the proposal was eventually carried. That conviction of sin was brought home to the Rev. John Clegg for his remark, really a disgraceful one for a man occupying his public and clerical position, and therefore presumably acquainted both with the elements of Christian charity and the simplest facts of life, is seen in that when Councillor Crooks said, with pointed good sense, "Would our reverend friend like to live on it?" Mr. Clegg's reply was, "That is an insolent remark." What else could he say, except apologise? Starving childhood is not true economy, as the clerk pointed out, and in case this argument should be insufficient by itself, we may remind Mr. Clegg that the Christian religion forbids the oppression of the fatherless and the widow, and the sending of the hungry empty away.

### A RED CROSS GRIEVANCE IN SCOTLAND.

Something more than the natural disappointment of those who have made preparations which are not made use of attaches to the grievance put forward by Mr. E. H. Robertson, president of the directors of the Forfar Infirmary, owing to the fact that the hospital arrangements for the reception of wounded and convalescent soldiers in the county have not been taken advantage of outside Dundee. In explaining the apparent neglect of the county at the quarterly meeting of infirmary directors last week, Mr. Robertson pointed out that there were three military hospital areas in Scotland—Glasgow, Edinburgh and Dundee—and four Red Cross areas. This, therefore, meant that Forfarshire came into the fourth or extra division, and that the numbers of wounded which could be allotted to it was comparatively small since there was no regular military hospital at Dundee. The Red Cross, he explained, had accommodation for some 350 patients in Dundee, and at present there were about 140; but if they went to Aberdeen they found there were more wounded than could be conveniently disposed of. The Commissioner of the Red Cross at Dundee had replied sympathetically to Mr. Robertson's suggestion that under these circumstances red tape might be relaxed, and the surplus wounded be sent to Forfar, Montrose, or Arbroath; but nothing had been done in the matter. This may, of course, have been due to the interference of recent weather conditions with the fighting in France, but with the replacement of impassable mud by frozen ground, or the coming spring weather, this cessation of action must shortly be replaced by active operations. If, therefore, there is indeed a surplus of wounded at Aberdeen, or anywhere else for that matter, it is essential that they should be disposed of, and the places to which they should be sent are not new institutions, improvised by patriotic effort in the congested areas, but to those whose active preparations have not been made use of hitherto.

### TORQUAY HOSPITALS AND THE WOUNDED.

The Western Hospital for Consumption, which this season has been receiving wounded soldiers instead of its ordinary patients, has now provided an operating theatre and appliances to enable it to be in a better position to treat the soldiers. The whole of the cost, towards which £50 was given by the widow of the late Dr. Paul, one of its honorary physicians, who died during 1914, has been privately subscribed. Situated in its own grounds overlooking the sea, this hospital occupies a fine position. Sir Rudolph Hampden-Smith, Bart., will act as house surgeon during the war. In the case of the Torbay Hospital, Torquay, which at the present time contains wounded English and Belgian soldiers and a sailor from the North Sea Fleet, owing to the difficulty of obtaining a house surgeon, Dr. Allan Bennett, the honorary *x*-ray surgeon, is giving his services, and is living in the hospital. The institution appears to have had a very successful year financially, and, although the expenses increased by over £400, sufficient money was received not only to pay its way, but to clear off the debt of nearly £200 from the previous year. In this connection, and as an encouragement to other towns, it should be noted that the Mayor of Torquay opened a fund to help the various institutions in the town engaged in treating wounded men, and he has received up to date the sum of £1,620.

### AN IMPROVEMENT IN THE ANNUAL REPORT.

Three interesting points with reference to hospital policy are brought out in the report to the Court of Contributors by the committee appointed to consider the managers' report of the Edinburgh Royal Infirmary—namely, as regards wounded soldiers, insured patients, and the Inland Revenue. While approving of the advantages of the Royal Infirmary being generously offered to the wounded, the committee look upon their treatment as part of the cost of the war, and suggest that the managers should put the case for recovering payment from the Government for the wounded before the authorities. In respect to the treatment of insured persons the committee again point out that the voluntary hospitals by providing the institutional treatment omitted from the Insurance Act have made its working possible, and are entitled to be recouped for the expenditure already, and in the future, incurred. In line with the arguments which The Hospital has already published on the subject the committee urge that application should be made to the Inland Revenue authorities to remit the duty on alcohol used in hospitals in the making of tinctures, galenicals, etc., and they are informed that the present duty represents some nine-tenths of the total cost of the alcohol so used. The above is of interest apart from its details as representing, it will be noticed, a complete hospital policy in little, clearly and tersely put forward as a kind of digest for the Court of Contributors of the managers' report. Such a means of consolidating a body of public opinion, informed on the principal points and problems engaging the managers of a hospital each year, is no doubt only fully possible to a great institution, like the Edinburgh Royal Infirmary, with a large body of supporters behind it. But the principle involved is one which we think might well be taken advantage of, for it resolves itself, at bottom, into an attempt to make a hospital report, on broad lines, intelligible and interesting to the rank and file of hospital supporters and the general public. The number of patients, attendances, operations, income and expenditure accounts: these necessary statistics should be supplemented by an essay on the work and policy of the institution, separately presented, if need be, as in this case, but anyway in more intelligible fashion than the perfunctory paragraphs which usually begin a typical annual report.

### PUBLIC GARDENS AS A MILITARY HOSPITAL.

The Cardiff Parks Committee recently received Colonel Hepburn, Commanding Officer, and Major E. Maclean, Adjutant and Registrar, representing the 3rd Western General Hospital, who requested their permission to take over Howard Gardens, the road between them, and the Higher Grade School for the erection of new hospital buildings for wounded soldiers. By this means the present accommodation of 540 beds, which includes ninety-four at King Edward VII.'s Hospital, would be increased to some 1,100 beds, and thereby enable the authorities to deal with the greater number of cases which are expected. The gardens, containing walks, tennis courts, and bowling green, are set apart for the use of residents, and it is proposed to cover them with temporary buildings and to utilise half of the school yards for the erection of wards, and the remainder of the playing space for convalescents. Colonel Hepburn pointed out that local tradespeople benefited by the presence of the military hospitals, as no attempt was made to dump supplies from a distance, and that the tennis courts would be covered by buildings on the pavilion system of a decorative kind, so as to be no disfigurement to the locality. He also stated that the War Office would bear all the cost, and would replace the gardens in the same condition when the need for accommodation ceased. It would be necessary to take over the roadway in order to connect the existing hospital with the new buildings, but it was not anticipated that any difficulty would arise with the Public Works Committee. The Parks Committee granted permission to proceed with the scheme, subject to the Town Clerk stating that there would be no legal barrier.

### THE MANUFACTURE OF SYNTHETIC DRUGS AND ANILINE DYES.

From the point of view of medical men and pharmacists, the proposed national scheme for the establishment of the coal-tar colour industry on a large and permanent basis in this country is of interest mainly because of the close relation between aniline dyes and synthetic drugs. In German factories the two branches of this wonderful industry—colours and drugs—are conducted side by side, the chemistry of the two being so nearly allied. It is therefore inconceivable that the production of aniline dyes will be prosecuted on an extensive scale in Great Britain, and the sister industry—the manufacture of drugs of the coal-tar series—neglected. It has only to be recalled that when Perkin invented the first aniline dye he was not conducting his researches with that object in view, but was attempting to synthesise quinine, to show how the work done in the research laboratories of a great coal-tar colour factory may lead to discoveries of new drugs. For these reasons alone it is disappointing to hear rumours that there is a possibility of the Government scheme falling through for lack of support from private capital, and it is to be hoped that it will be possible to modify the plans sufficiently to make the scheme acceptable to all parties. In recent years the most valuable additions to materia medica have emanated from factories whose business consisted largely in the making of artificial colouring matter, and it would be a stupendous misfortune if our manufacturers were to neglect the present opportunity of breaking the German monopoly in drugs of this series.

### THE NEW MANCHESTER UNION.

The Local Government Board, after a year's interval since their approval of the principle of amalgamation was given, issued their Order last week for the dissolution of the Prestwich Poor-Law Union and for the creation of a new Manchester Union, by which all the city districts will be embraced, and under which, for Poor-Law purposes, Prestwich and Failsworth will be included. The duties of the new Union will start on April 1, and the number of Guardians will be then reduced from the sixty-two now elected for the three boards to forty. The smallest number of Guardians elected will be, according to the new Order, two, for Failsworth; and the largest seventeen, for South Manchester. The views of the different boards will soon be available, but no doubt some of them will criticise the reduced figure. The object of the change is, of course, to increase efficiency by uniformity of administration, and incidentally also to secure financial equity, which has not hitherto obtained. We have before now in London seen so much benefit arising from the partial growth of centralisation between the different authorities that, though it is very far from being completed in the Metropolis, where different authorities create much overlapping, the official formation of the new Manchester Union is warmly to be welcomed. It is another step on the road of unified control and centralised administration, under which alone the Poor-Law infirmaries and the Poor-Law Medical Service can be properly co-ordinated, as we have so often pointed out. Indeed, the time is ripe for more centralised control, since the staff difficulty, now so great, can be better coped with by one central body than by several isolated boards.

### THIS WEEK'S DRUG MARKET.

Business continues to improve, and in the prices of a number of articles there is a distinctly upward tendency. Quinine is in fair demand, and the probability of a rise in price is spoken of; it is, of course, difficult to foresee what will happen, but in view of the plentiful supplies of quinine sulphate in this country it hardly seems likely that the advance, if any, would be a substantial one. Cod-liver oil still shows a tendency to advance, but the present demand is by no means large. Several synthetic drugs, including acetanilide and barbitone, are dearer; the upward movement in the value of salicylic acid and the salicylates seems likely to continue as a result of the shortage of supplies; the high price of carbolic acid has, of course, increased the cost of production of salicylic acid, and this is a factor which must be taken into account, as well as the stoppage of supplies of salicylates from Germany. Belladonna root and atropine continue to tend upwards in price. The price of cocaine is well maintained. The demand for morphine and codeine continues strong. Cream of tartar has a higher tendency in price. At the recent public sale of drugs in Mincing Lane senna leaves fetched substantially higher prices, but otherwise the demand for the drugs offered was small.

